# Prehospital randomised assessment of a mechanical compression device in cardiac arrest (PaRAMeDIC) trial protocol

**DOI:** 10.1186/1757-7241-18-58

**Published:** 2010-11-05

**Authors:** Gavin D Perkins, Malcolm Woollard, Matthew W Cooke, Charles Deakin, Jessica Horton, Ranjit Lall, Sarah E Lamb, Chris McCabe, Tom Quinn, Anne Slowther, Simon Gates, PARAMEDIC trial collaborators

**Affiliations:** 1 Warwick Clinical Trials Unit, Warwick Medical School, University of Warwick, Gibbet Hill Road, Coventry CV4 7AL, UK; 2 Pre-hospital, Emergency and Cardiovascular Care Applied Research Group, Coventry University, Priory Street, Coventry, CV1 5FB, UK; 3 Warwick Medical School, University of Warwick, Coventry, CV4 7AL, UK; 4 Dept of Anaesthetics, Southampton General Hospital, Tremona Road, Southampton, SO16 6YD, UK; 5 Academic Unit of Health Economics, Leeds Institute of Health Sciences, Charles Thackrah Building, University of Leeds, 101 Clarendon Road, Leeds, LS2 9LJ, UK; 6 23DK04, Duke of Kent Building, Division of Health and Social Care, Faculty of Health and Medical Sciences, University of Surrey, Guildford, UK, GU2 7XH; 7 University of Warwick, Medical Teaching Building, University of Warwick, Coventry, CV4 7AL, UK

## Abstract

**Background:**

Survival after out-of-hospital cardiac arrest is closely linked to the quality of CPR, but in real life, resuscitation during prehospital care and ambulance transport is often suboptimal. Mechanical chest compression devices deliver consistent chest compressions, are not prone to fatigue and could potentially overcome some of the limitations of manual chest compression. However, there is no high-quality evidence that they improve clinical outcomes, or that they are cost effective. The Prehospital Randomised Assessment of a Mechanical Compression Device In Cardiac Arrest (PARAMEDIC) trial is a pragmatic cluster randomised study of the LUCAS-2 device in adult patients with non-traumatic out-of-hospital cardiac arrest.

**Methods/design:**

The primary objective of this trial is to evaluate the effect of chest compression using LUCAS-2 on mortality at 30 days post out-of-hospital cardiac arrest, compared with manual chest compression. Secondary objectives of the study are to evaluate the effects of LUCAS-2 on survival to 12 months, cognitive and quality of life outcomes and cost-effectiveness. Methods: Ambulance service vehicles will be randomised to either manual compression (control) or LUCAS arms. Adult patients in out-of-hospital cardiac arrest, attended by a trial vehicle will be eligible for inclusion. Patients with traumatic cardiac arrest or who are pregnant will be excluded. The trial will recruit approximately 4000 patients from England, Wales and Scotland. A waiver of initial consent has been approved by the Research Ethics Committees. Consent will be sought from survivors for participation in the follow-up phase.

**Conclusion:**

The trial will assess the clinical and cost effectiveness of the LUCAS-2 mechanical chest compression device. Trial Registration: The trial is registered on the International Standard Randomised Controlled Trial Number Registry (ISRCTN08233942).

## Background

Sudden cardiac death is a major cause of death and morbidity in the Western world. In Europe, approximately 700,000 people sustain a cardiac arrest in the community each year[1,2]. Resuscitation is attempted in about 45% of cases of which approximately 20% achieve a return of spontaneous circulation by the time of arrival at hospital and about 5% survive to hospital discharge[3,4]. Good quality cardiopulmonary resuscitation (CPR) has a significant impact on the likelihood of survival[5-7], yet it is difficult to perform in the prehospital environment due to the multiple tasks required upon arrival at a cardiac arrest. In addition, rescuer fatigue can reduce chest compression quality as early as 1 minute after commencing chest compressions[8].

The LUCAS-2 is a mechanical device that delivers sternal compressions at a constant rate of 100 per minute, to a fixed depth of 4 cm to 5 cm, using a piston with a suction cup attached that returns the chest to its normal expanded position. The rate and depth comply with International scientific guidelines on CPR[9]. It is easy to apply, stable in use, relatively light in weight (7.8 kg), and well adapted to use during patient movement on a stretcher and during ambulance transportation. The device is CE marked and has been on the market since 2002 in Europe. The device was originally gas-powered, a battery powered version (LUCAS-2) was introduced in 2009. Detailed descriptions of the device and experimental data from animal studies reporting increased cardiac output and cortical cerebral flow compared to manual standardised CPR have been published[10]. However there is a lack of robust evidence from human trials for the clinical and cost effectiveness of the device [11,12].

The Prehospital Randomised Assessment of a Mechanical Compression Device In Cardiac Arrest (PARAMEDIC) trial is a cluster randomised pragmatic trial of the clinical and cost effectiveness of the LUCAS-2 device versus manual chest compression, for adult patients in whom resuscitation is attempted following non-traumatic out-of-hospital cardiac arrest.

## Methods/Design

### Trial Approvals and Conduct

The trial is approved by the Coventry Research Ethics committee (for England and Wales) and Scotland A Research Ethics Committee. The trial is registered on the International Standard Randomised Controlled Trial Registry (ISRCTN08233942). It will be carried out in accordance with the Medical Research Council (MRC) Good Clinical Practice Guidelines[13], applicable UK legislation and the Standard Operating Procedures of the Warwick Clinical Trials Unit. The sponsor organisation for the trial is the University of Warwick. The trial is funded by the National Institute for Health Research (NIHR) Health Technology Assessment (HTA) Programme[14] and is a collaboration between the Universities of Warwick, Coventry, Leeds, Southampton and Surrey and the West Midlands, Scottish and Welsh NHS Ambulance Services. Further details can be found on the trial website[15].

The contribution of the manufacturers (JOLIFE AB) and distributors (Physio-Control UK) of the LUCAS-2 device will be limited to supply and servicing of LUCAS-2 devices, and training of study co-ordinating centre personnel. They will have no role in the design, conduct, analysis or reporting of the trial.

### Outcome Measures

The primary outcome for the trial is survival to 30 days post cardiac arrest. Secondary outcomes are: survival of event (sustained return of spontaneous circulation (ROSC) to arrival at hospital); survival to hospital discharge and to 12 months; health related quality of life at 3 and 12 months (measured by SF12 and EQ-5D); neurologically intact survival to 3 months (survival with Cerebral Performance Category (CPC) score 1 or 2); cognition at 12 months (Mini Mental State Examination (MMSE); anxiety and depression at 12 months (Hospital Anxiety and Depression Scale (HADS)); post traumatic stress at 12 months (PTSD civilian checklist (PCL-C)); hospital length of stay; intensive care length of stay.

### Eligibility Criteria

Vehicles that are in service at participating ambulance stations and may attend cardiac arrests will be included in the trial, and will randomised before recruitment starts to either the LUCAS or manual chest compression (control) arms. To maximise the efficiency of the trial, recruitment will be predominantly concentrated in urban areas.

Individual patients will be eligible if:

1. they are in cardiac arrest in the out-of-hospital environment on arrival of a trial vehicle;

2. the first ambulance resource to arrive is a trial vehicle

3. a resuscitation attempt is initiated by the attending paramedic, according to UK national guidelines;

4. the patient is known or believed to be aged 18 years or over.

Exclusion criteria will be:

1. traumatic cardiac arrest

2. known or clinically apparent pregnancy.

Treatment allocation of each individual participant will be determined by the first trial vehicle to arrive on scene. If this is a LUCAS trial vehicle, the patient will be included in the LUCAS arm, and if it is a control trial vehicle, the patient will be in the control arm. If a non-trial ambulance or rapid response vehicle arrives first and resuscitation is started, the patient will be excluded.

### Power and Sample Size

There are no national data on survival to 30 days post cardiac arrest. However, it is likely to be very similar to survival to hospital discharge, as most mortality will occur in the period immediately following a cardiac arrest. In a systematic review[1], the average survival to hospital discharge in 8 studies conducted in the UK was 8.1%. National audit data for England (2004-2006) indicate that the proportion of patients with ROSC at hospital admission is 14 to 16%[4]. Estimates of mortality in hospital vary from 50% to 70%, hence the incidence of survival to hospital discharge is expected to be between 4.5% and 8%[16]. A conservative estimate of survival to 30 days is therefore 5%.

No data currently exist from which a relevant intracluster correlation coefficient (ICC) can be calculated, and we have therefore assumed a conservative value of 0.01. The value of the ICC will be monitored at interim analyses by the Data Monitoring Committee (DMC), who will make recommendations for adjustments to the required sample size.

### Sample Size Required

**Figure 1:**
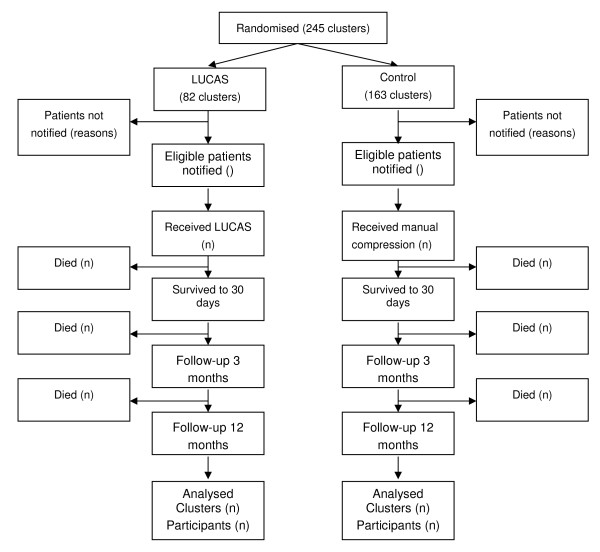
**Flow chart for PARAMEDIC Trial**.

We aim to detect, with 80% power, an increase in the incidence of survival to 30 days from 5% in the control arm to 7.5% in the LUCAS arm (a risk ratio of 1.5). The number of LUCAS clusters (vehicles) is limited by the number of devices available, but because control clusters (vehicles) do not require any specific equipment, we can include more control clusters than LUCAS clusters in the trial (see figure 1). Detection of the specified difference with a randomisation ratio of 1:2 and a cluster size of 15 requires 82 LUCAS and 163 control clusters (3675 participants in total). The primary outcome will be determined for close to 100% of trial participants, so there is no adjustment for losses of individual patients.

### Consent

Prospective consent from research participants prior to enrolment is impossible in this trial; the occurrence of an out-of-hospital cardiac arrest is unpredictable, and a victim becomes unconscious within seconds. Treatment (in the form of CPR) must be started immediately in an attempt to save the person's life. It is therefore not practical to consult a carer or independent clinician without causing the potential participant harm as a result of delaying treatment. Conducting research in emergency situations where a patient lacks capacity is regulated by the Mental Capacity Act (2005) in England and Wales and the Adults with Incapacity Act (2000) in Scotland. The relevant ethics committees have determined that the research methods are compliant with the requirements of this legislation.

Consent for follow-up will be sought from all participants who survive to hospital discharge. If a participant lacks capacity to give informed consent we will seek the views of a personal consultee in order to establish the patient's wishes.

### Protection against Bias

#### Cluster design

Selection bias is a major potential problem in cluster randomised trials: patients with different characteristics may be recruited to the two trial arms[17]. Further bias can arise where a large proportion of eligible patients are not included in the trial, as the probability of inclusion may be related to the intervention. In this trial we will identify eligible patients from routinely collected ambulance service data, which will allow us to identify and include close to 100% of the eligible patients, thus avoiding selection bias.

#### Threshold for resuscitation

Paramedics need to make a rapid decision as to whether to resuscitate someone in cardiac arrest upon arrival at the scene. It is possible that application of the Recognition of Life Extinct (ROLE) criteria[18] will differ between the trial arms. If paramedics believe strongly that LUCAS-2 is effective, some of them may attempt to resuscitate patients in the LUCAS arm who have no chance of survival, and for whom a resuscitation attempt would not normally be considered. This would result in a group of patients with very low probability of survival being recruited to the LUCAS arm but not the control arm, potentially masking any beneficial effect of LUCAS-2.

We will monitor the accumulating trial data for evidence of a between-group difference in threshold as follows: (1) proportion of arrests where resuscitation attempted versus cardiac arrests attended (2) patient age profile (3) proportion receiving bystander CPR (4) time from collapse to trial vehicle arrival and (5) proportion of patients in asystole. If evidence of bias is detected corrective action will be taken.

#### Compliance

Compliance (whether LUCAS-2 was used for all eligible patients in the LUCAS-2 arm and none in the control arm) will be monitored by review of ECG recordings taken during resuscitation. Recorded compression waveforms will be analysed to determine whether LUCAS-2 was used and to confirm the presenting rhythm and duration of resuscitation.

#### Learning effects

Because LUCAS-2 will be new to paramedics in the areas where the trial is conducted, there is a possibility that there will be a learning effect, and its apparent effectiveness may increase through time as personnel become more familiar with its use. We will therefore allow a "run-in" period before the start of recruitment to the trial at each station. Participating vehicles will be randomised at the start of this period, LUCAS-2 will be used in the LUCAS arm, and trial data will be collected but will not be included in the main trial analysis.

#### Crew preferences

With randomisation by vehicle, a potential source of bias is that paramedics who are motivated to use LUCAS-2 will select LUCAS vehicles, whilst those who dislike the device may avoid it. In order to check for this possibility, we will review records of crews members present at each cardiac arrest to check individuals who consistently appear in one arm. If swapping between LUCAS and control trial vehicles is found to occur, the staff involved will be given extra training in the trial procedures.

#### Blinding

Because of the nature of the interventions, paramedics cannot be blinded, and will be aware of treatment allocations. Control room personnel will be blinded to the allocation of the ambulance service vehicles, to ensure that there is no bias in whether a LUCAS or control trial vehicle is sent to an incident that is likely to be a cardiac arrest. Patients themselves will be unaware of their treatment allocation at the time of the intervention, though they may subsequently be unblinded by relatives or friends who are aware that LUCAS-2 was used. To ensure blinding of outcome assessment as far as possible, research nurses assessing patients at follow-up visits will be blinded to the allocated treatment group.

### Trial Intervention/Treatments

#### LUCAS arm

The trial will use the LUCAS-2 device, (JOLIFE AB, Ideon Science Park, Scheelevägen 17, SE-223 70 Lund, Sweden).

**Figure 2:**
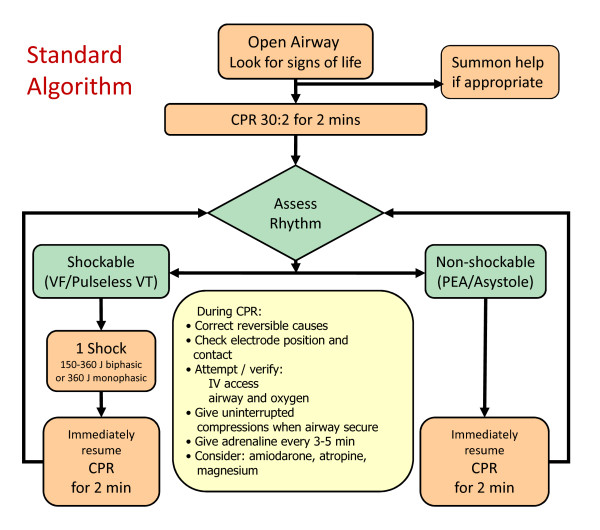
**Treatment algorithm for control arm**.

**Figure 3:**
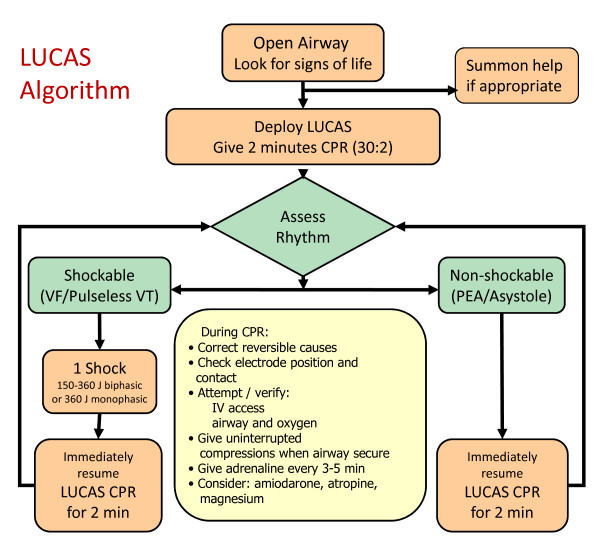
**Treatment algorithm for LUCAS arm**.

The LUCAS arm will receive resuscitation according to the Resuscitation Council (UK)[19] and Joint Royal College Ambulance Liaison Committee (JRCALC) advanced life support guidelines[20] with the exception that the LUCAS-2 device will be deployed to replace manual chest compressions (see figures 2 and 3). All standard advanced life support interventions will be provided including drug administration, defibrillation and advanced airway management as required.

On arrival, two minutes of LUCAS-2 CPR (5 cycles of 30:2) will be administered before a countershock if the patient is in ventricular fibrillation (VF) or pulseless ventricular tachycardia (VT). Operational experience shows that LUCAS-2 can be positioned and activated within 20 to 30 seconds of arrival at the patient. Prior to intubation, compressions will be provided using the 30 compressions to 2 ventilation mode. If the patient is intubated, asynchronous compressions and ventilations will be provided, with a ventilation rate of 10 per minute. A bag-valve device will be used to manually provide ventilations.

Defibrillation will be performed using the following sequence: stop LUCAS-2 device, analyse heart rhythm; if shock indicated, restart LUCAS-2, charge, deliver shock, continue CPR for 2 minutes. This will minimize deleterious pre and post shock pauses in compressions. The LUCAS-2 device will be used in place of standard chest compressions as long as continued resuscitation is indicated, including outside the ambulance and during transport to hospital. The trial intervention will cease after care is handed over to the medical team at hospital.

If a patient in the LUCAS arm arrives at hospital with the LUCAS-2 device running, the device should be removed and resuscitation should continue with manual compressions. Hospitals will be given information about the trial prior to the start of recruitment.

#### Manual chest compression arm

The control arm will receive resuscitation according to the Resuscitation Council (UK) and JRCALC Advanced Life Support Guidelines.

#### Guidelines change in 2010

The International Liaison Committee for Resuscitation and European Resuscitation Council (UK) will publish new resuscitation guidelines on 18^th ^October 2010. There is likely to be a delay before these are incorporated into clinical practice. The LUCAS-2 and manual chest compression protocols will be updated to coincide with the adoption of the new guidelines in the respective ambulance services. A subgroup analysis will be undertaken to compare treatment effects of LUCAS-2 before and after introduction of the new guidelines.

### Data Collection

Data up to admission to hospital will be extracted from routinely collected ambulance service data, and will be supplied to the trial database in anonymised form. Local Register Offices will be contacted by ambulance services after each individual's cardiac arrest, to verify whether the participant is alive and to ensure that communications about participation in the follow-up are not sent to deceased individuals. If patients have died, the date and location of death will be recorded. Trial participants will be flagged on the NHS Central Register so that later deaths will be notified to the trial.

#### Follow-up

Where consent is given, surviving participants will be followed up approximately 3 months after their cardiac arrest, by a home visit from a study research nurse or paramedic. At this visit the quality of life measures will be completed, details of ICU and hospital discharge dates will be collected, and an assessment of CPC score made.

The second follow-up visit at 12 months will include quality of life, anxiety and depression (HADS), post-traumatic stress (PCL-C) and Mini-Mental State Examination (MMSE). Health service and social care resource use will be recorded by participants at the 3 month and 12 month follow-up.

#### Serious Adverse Events (SAEs) and Serious Adverse Device Effects (SADEs)

SAEs and SADEs will be reported to the trial co-ordinating centre if they fulfil the criteria for seriousness, they are potentially related to trial participation, and they are unexpected i.e. the event is not an expected occurrence for patients who have had a cardiac arrest.

### Statistical Analysis

All analyses will be by intention to treat, and all estimates will be adjusted to account for the cluster randomised design. Dichotomous outcomes (survival to 30 days, hospital discharge, 3 months and 12 months, and neurologically intact survival) will be presented as risk ratios and 95% confidence intervals. Survival will also be analysed as a time to event outcome, using survival analysis, with adjustment for clustering and important covariates. Results will be presented using hazard ratios and their 95% confidence intervals. Other time to event outcomes (duration of hospital and ICU stay) will be analysed in the same way. Continuous outcomes (quality of life, anxiety and depression, cognition and post traumatic stress) will be analysed by multi-level linear regression, with adjustments for important covariates. The results will be presented as the difference in means between the groups and its 95% confidence interval. CPC score will be analysed by multi-level ordinal logistic regression[21] and the results will be presented using odds ratios and their 95% confidence intervals. Reporting of analyses will follow CONSORT guidelines for the reporting of cluster randomised trials[22]. A detailed analysis plan will be drawn up by the study statisticians and approved by the DMC.

Four pre-specified subgroup analyses will be conducted to conform with Utstein recommendations: witnessed cardiac arrest versus not witnessed; bystander CPR versus no bystander CPR; type of initial rhythm (VF/VT; PEA; asystole); presumed cardiac aetiology of cardiac arrest. Subgroup analyses will use statistical tests of interaction [23]. In addition, we will model the effects of age and the time interval from the 999 call to arrival of the trial vehicle on the effects of the LUCAS-2 intervention, using logistic regression analyses.

Interim analyses will be conducted at least once per year during recruitment and supplied confidentially to the DMC, who will consider the results and make recommendations to the Trial Steering Committee (TSC) about continuation of recruitment or any modification to the trial that may be necessary.

### Economic analysis

An economic evaluation will be conducted alongside the trial, consisting of a within-trial cost effectiveness analysis, comparing the observed costs and outcomes of the intervention and control groups during the trial period, and analysis of the long-term incremental cost effectiveness of LUCAS-2, by constructing a decision analytic cost effectiveness model with a lifetime horizon.

For the within trial economic evaluation the interventions (LUCAS-2 vs. manual compression) will be compared in terms of Quality Adjusted Life Years (QALYs). The utility weights for calculating the QALYs will be derived from the EQ-5 D and SF-12 [24] via the SF-6 D algorithm[25]. The outcomes will be reported as the expected incremental cost effectiveness of LUCAS-2- compared to usual care.

## Conclusion

There remains an urgent need to improve outcomes from cardiac arrest. The quality of CPR is known to significantly influence outcomes from cardiac arrest but despite this, in real life it is often performed suboptimally. Mechanical chest compression devices may overcome some of the limitations of manual CPR, yet there is a paucity of high quality clinical evidence to support their use. The PARAMEDIC trial is a large, multi-centre, pragmatic trial aiming to evaluate the clinical and cost effectiveness of the LUCAS-2 mechanical chest compression device in out-of-hospital cardiac arrest.

## List of abbreviations

CONSORT: Consolidated Standards of Reporting Trials; CPR: Cardiopulmonary resuscitation; DMC: Data monitoring committee; EQ-5 D: EuroQol 5 Dimensions; HADS: Hospital Anxiety and Depression Scale; JRCALC: Joint Royal College Ambulance Liaison Committee; LUCAS-2: Lund University Cardiopulmonary Assistance System; MMSE: Mini Mental Health State Exam; OHCA: Out-of-hospital cardiac arrest; PEA: Pulseless electrical activity; QALYs: Quality Adjusted Life Years; ROSC: Return of spontaneous circulation; ROLE: Recognition of life extinction; SAE: Serious adverse event; SF-12: Short form-12; TSC: Trial Steering Committee; VF: Ventricular Fibrillation; VT: Ventricular Tachycardia.

## Competing interests

The authors declare that they have no competing interests.

## Authors' contributions

GDP and SG are co-chief investigators for the trial. The trial protocol was developed by the authors of this paper. GDP/SG prepared the first draft of this summary protocol paper and revised in light of comments from co-investigators. All authors approved the final version of the paper.

## Author information

GDP is an Associate Clinical Professor in Critical Care and Resuscitation. He is a member of the European and UK Resuscitation Councils. SG is a Principal Research Fellow at the Warwick Clinical Trials Unit specialising in clinical trials and statistical methods in medical research.

## References

[B1] Atwood C Eisenberg MS Herlitz J Rea TD Incidence of EMS-treated out-of-hospital cardiac arrest in Europe Resuscitation 2005 67 1 75 80 10.1016/j.resuscitation.2005.03.021 16199289

[B2] Berdowski J Berg RA Tijssen JG Koster RW Global incidences of out-of-hospital cardiac arrest and survival rates: Systematic review of 67 prospective studies Resuscitation 2010 81 11 1479 1487 10.1016/j.resuscitation.2010.08.006 20828914

[B3] London Ambulance Service Cardiac Arrest Annual Report 2008/9 http://www.londonambulance.nhs.uk/about_us/idoc.ashx?docid=498ba02b-466b-48be-8d4d-7dcc3c38bfd0&version=-1

[B4] Ambulance Service Association and Joint Royal College Ambulance Liaison Committee National Cardiac Arrest Audit Report 2006

[B5] Wik L Steen PA Bircher NG Quality of bystander cardiopulmonary resuscitation influences outcome after prehospital cardiac arrest Resuscitation 1994 28 3 195 203 10.1016/0300-9572(94)90064-7 7740189

[B6] Edelson DP Abella BS Kramer-Johansen J Wik L Myklebust H Barry AM Merchant RM Hoek TL Steen PA Becker LB Effects of compression depth and pre-shock pauses predict defibrillation failure during cardiac arrest Resuscitation 2006 71 2 137 145 10.1016/j.resuscitation.2006.04.008 16982127

[B7] Kramer-Johansen J Myklebust H Wik L Fellows B Svensson L Sorebo H Steen PA Quality of out-of-hospital cardiopulmonary resuscitation with real time automated feedback: a prospective interventional study Resuscitation 2006 71 3 283 292 10.1016/j.resuscitation.2006.05.011 17070980

[B8] Hightower D Thomas SH Stone CK Dunn K March JA Decay in quality of closed-chest compressions over time Annals of Emergency Medicine 1995 26 3 300 303 10.1016/S0196-0644(95)70076-5 7661418

[B9] International Liaison Committee on Resuscitation 2005 International Consensus on Cardiopulmonary Resuscitation and Emergency Cardiovascular Care Science with Treatment Recommendations. Part 2: Adult basic life support Resuscitation 2005 67 2-3 187 201 10.1016/j.resuscitation.2005.09.016 16324988 PMC7144408

[B10] Steen S Liao Q Pierre L Paskevicius A Sjoberg T Evaluation of LUCAS, a new device for automatic mechanical compression and active decompression resuscitation Resuscitation 2002 55 3 285 299 10.1016/S0300-9572(02)00271-X 12458066

[B11] Perkins GD Brace S Gates S Mechanical chest-compression devices: current and future roles Curr Opin Crit Care 2010 16 3 203 210 10.1097/MCC.0b013e328339cf59 20463463

[B12] Jacobs I Mechanical chest compression devices--will we ever get the evidence? Resuscitation 2009 80 10 1093 1094 10.1016/j.resuscitation.2009.08.003 19699022

[B13] Medical Research Council MRC guidelines for good clinical practice in clinical trials 1998 London: Medical Research Council

[B14] A randomised controlled trial of the LUCAS mechanical compression/decompression device for out of hospital cardiac arrest. [LUCAS-UK] http://www.hta.ac.uk/project/1845.asp

[B15] Paramedic trial website http://www.warwick.ac.uk/go/paramedic

[B16] Pell JP Sirel J Marsden AK Cobbe SM Seasonal variations in out of hospital cardiopulmonary arrest Heart 1999 82 6 680 683 10573492 10.1136/hrt.82.6.680 PMC1729204

[B17] Eldridge S Kerry S Torgerson DJ Bias in identifying and recruiting participants in cluster randomised trials: what can be done? BMJ 2009 339 b4006 10.1136/bmj.b4006 19819928

[B18] Recognition of Life Extinct by Ambulance Clinicians http://www2.warwick.ac.uk/fac/med/research/hsri/emergencycare/prehospitalcare/jrcalcstakeholderwebsite/guidelines/recognition_of_life_extinct_by_ambulance_clinicians_2006.pdf

[B19] Deakin C Nolan JP Perkins GD Handley A Advanced Life Support Guidelines for the UK Resuscitation Guidelines for the UK 2005 Resuscitation Council (UK)

[B20] JRCALC Advanced cardiac life support guidelines http://www2.warwick.ac.uk/fac/med/research/hsri/emergencycare/prehospitalcare/jrcalcstakeholderwebsite/guidelines/adult_advanced_life_support_als_2006.pdf

[B21] Calculation of sample size for stroke trials assessing functional outcome: comparison of binary and ordinal approaches Int J Stroke 2008 3 2 78 84 10.1111/j.1747-4949.2008.00184.x 18705999

[B22] Campbell MK Elbourne DR Altman DG group C CONSORT statement: extension to cluster randomised trials.[see comment] BMJ 2004 328 7441 702 708 10.1136/bmj.328.7441.702 15031246 PMC381234

[B23] Altman DG Bland JM Interaction revisited: the difference between two estimates.[see comment] BMJ 2003 326 7382 219 10.1136/bmj.326.7382.219 12543843 PMC1125071

[B24] Ware JE Kolinski M Keller SD How to Score the SF-12 Physical and Mental Health Summaries: A User's Manual 1995 Boston, MA: The Health Institute, New England Medical Centre

[B25] Brazier JE Roberts J The estimation of a preference-based measure of health from the SF-12 Medical Care 2004 42 9 851 859 10.1097/01.mlr.0000135827.18610.0d 15319610

